# The ubiquitination–autophagy axis in cancer therapy resistance: mechanistic insights and therapeutic opportunities

**DOI:** 10.3389/fphar.2025.1722559

**Published:** 2026-01-21

**Authors:** Hengrui Zhang, Hanxi Yan, Yulin Liu, Anqi Zeng, Linjiang Song

**Affiliations:** 1 School of Medical and Life Sciences, Chengdu University of Traditional Chinese Medicine, Chengdu, China; 2 Translational Chinese Medicine Key Laboratory of Sichuan Province, Sichuan Academy of Chinese Medicine Sciences, Sichuan Institute for Translational Chinese Medicine, Chengdu, Sichuan, China

**Keywords:** autophagy, chemotherapy, E3 ligase, immune therapy, radiotherapy, targeted therapy, therapy resistance, ubiquitination

## Abstract

Therapy resistance is a major challenge in cancer treatment. Growing evidences reveal that the interaction between ubiquitination and autophagy plays a key role in regulating resistance to chemotherapy, radiotherapy, targeted therapy, and immunotherapy. In this review, we systematically summarize recent studies that reveal how specific E3 ligases, deubiquitinating enzymes, and ubiquitin-like modifiers influence autophagic flux and modulate the tumor response. We focus on key regulatory circuits—such as the Tripartite-motif protein 65–miR-138-5p–Autophagy related 7 (TRIM65–miR-138-5p–ATG7)pathway in non-small cell lung cancer, the Cullin-RING Ligase 4(CRL4)–mitophagy signaling pathway in ovarian cancer, and the Ubiquitin Specific Peptidase 14–S-phase kinase-associated protein 2(USP14–Skp2) axis in B-Raf proto-oncogene (BRAF) inhibitor resistance—illustrating the dual regulatory functions of ubiquitin-dependent protein turnover and autophagy. Furthermore, we highlight how noncoding RNAs and the tumor microenvironment influence ubiquitination-modulated autophagy and contribute to immune resistance or DNA repair remodeling. Finally, we discuss potential therapeutic strategies, including Proteolysis Targeting Chimeras (PROTACs), dual E3 ligase/autophagy inhibitors, and autophagy flux modulators, to overcome resistance and enhance treatment efficacy across multiple cancer types. These insights establish the foundation for targeting the ubiquitin–autophagy network as a cohesive strategy to combat refractory cancer.

## 1 Introduction

Cancer is still one of the main causes of illness and death worldwide. In 2018, the global incidence exceeded 18 million cases and is projected to reach nearly 29 million by 2040. The increase will be greater in developing and transitioning countries because of aging populations and lifestyle-related risk factors (Sung et al., 2021). Both ubiquitination and autophagy play key roles in these processes. Abnormal ubiquitination changes the stability of oncogenes and tumor suppressors, while dysregulated autophagy gives tumor cells metabolic flexibility and stress resistance (Wu et al., 2024; Ling et al., 2025). Together, the ubiquitination–autophagy axis forms an important regulatory network that drives tumor growth and shapes malignant behavior.

Post-translational modifications (PTMs) are key mechanisms that control protein function and help cells respond quickly to changes inside and outside the cell (Lee et al., 2023; Sharma et al., 2019). Among these modifications, ubiquitination is one of the most multifunctional and tightly regulated types. It works through a series of enzymes—E1 (ubiquitin-activating enzyme), E2 (ubiquitin-conjugating enzyme), and E3 (ubiquitin ligase). These enzymes attach ubiquitin to target proteins, which then controls many cellular processes, such as protein degradation, DNA repair, cell cycle control, and signal transduction (Gao et al., 2023; Ganesan and Kiyokawa, 2025). Dysregulation of ubiquitination disrupts protein homeostasis and signaling accuracy, broadly influencing cancer development (Sun et al., 2020; Liu F. et al., 2024). For instance, overexpression of Ubiquitin Specific Peptidase 24(USP24) in cancer cells has been shown to promote drug resistance, whereas pharmacological inhibition with the optimized compound USP24-i-101 reverses this effect by enhancing Programmed cell death ligand 1(PD-L1) degradation and maintaining genomic stability in an autophagy-dependent manner (Young et al., 2024).

Autophagy is an evolutionarily conserved lysosome-dependent degradation pathway that maintains cellular quality control by eliminating damaged organelles, misfolded proteins, and invading pathogens (Liu S. et al., 2023). Beyond its homeostatic role, autophagy is intricately involved in tumor biology and cancer therapy (Xia et al., 2021). Basal autophagy helps maintain genome stability and metabolic balance, which supports its tumor-suppressive role in the early stages of cancer development (Onorati et al., 2018; Jalali et al., 2025). However, once tumors are established, cancer cells frequently exploit autophagy to withstand stress induced by chemotherapy, targeted therapy, radiotherapy, and immunotherapy. By recycling intracellular components, autophagy enhances tumor cell survival, promotes therapy resistance, and contributes to disease progression (Hassan et al., 2024; Kwantwi, 2024). These context-dependent roles underscore autophagy as a double-edged sword in oncology, highlighting both its biological significance and its potential as a therapeutic target in overcoming cancer resistance.

Despite significant advances in cancer therapy, including chemotherapy, targeted therapy, radiotherapy, and immunotherapy, the emergence of therapeutic resistance remains a formidable clinical challenge (Gu et al., 2025; Wang H. et al., 2025). These limitations mainly come from an incomplete understanding of how tumors start, grow, and become resistant to treatment. Studies have revealed several overlapping resistance mechanisms, including cancer stem cell activity, DNA damage repair, and changes in the tumor microenvironment (Gu et al., 2025; Roszkowska, 2024). Therefore, a deeper insight into these physiological and pathological processes is essential for the development of more effective therapeutic strategies and the mitigation of treatment-associated complications.

**FIGURE 1:**
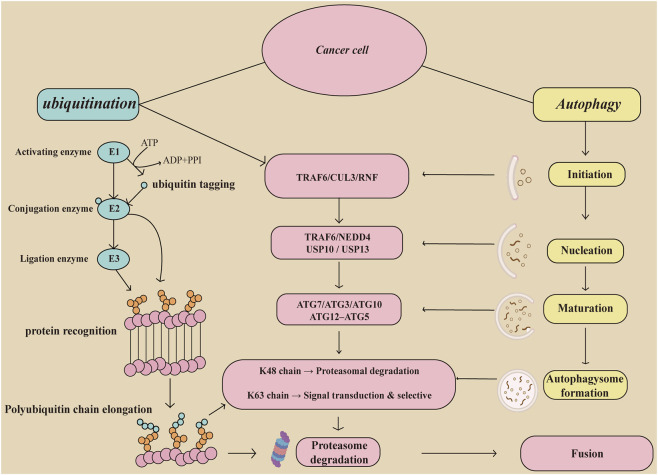
Overview of the molecular interplay between ubiquitination and autophagy in cancer cells. This schematic illustrates the crosstalk between ubiquitination and autophagy pathways in the context of cancer cell regulation. Ubiquitination is initiated by E1 (activating enzyme), E2 (conjugating enzyme), and E3 (ligase), leading to polyubiquitin chain elongation and protein tagging. The type of ubiquitin linkage determines the fate of substrates, for example: K48-linked chains signal proteasomal degradation, while K63-linked chains facilitate signal transduction and selective autophagy. Autophagy proceeds through distinct stages—initiation, nucleation, autophagosome formation, and fusion with lysosomes—culminating in degradation. Key autophagic regulators are modulated by ubiquitination, linking both processes through selective autophagy pathways. This diagram summarizes the functional integration of ubiquitin signaling with autophagic flux, a critical axis in tumor progression and therapeutic resistance. TRAF6,tumor necrosis factor receptor-associated factor 6; CUL3,Cullin 3; RNF,Ring Finger Protein; NEDD4,NEDD4 like E3 ubiquitin protein ligase; USP, Ubiquitin Specific Peptidase; ATG, Autophagy related 7.

This review provides an overview of how ubiquitination controls autophagy and contributes to cancer therapy resistance (Figure 1). We focus on four main treatment types: chemotherapy, targeted therapy, radiotherapy, and immunotherapy. In chemotherapy, examples include platinum-based drugs, doxorubicin, and paclitaxel. For targeted therapy, we discuss receptor tyrosine kinase inhibitors, immune checkpoint inhibitors, and other new molecular strategies. By summarizing recent findings on the ubiquitination–autophagy axis, this review explains the molecular basis of adaptive resistance, identifies possible therapeutic targets, and examines new approaches such as E3 ligase inhibitors, deubiquitinase inhibitors, and Proteolysis Targeting Chimeras (PROTACs)-based drugs. The goal is to highlight how targeting ubiquitination-driven autophagy can help overcome therapy resistance and improve outcomes for cancer patients.

## 2 Ubiquitin-mediated regulation of autophagy in chemotherapy resistance

### 2.1 Overview of ubiquitin–autophagy interaction in chemoresistance

Chemotherapy continues to represent a fundamental component of cancer treatment strategies. However, tumors frequently develop resistance through various mechanisms, including the upregulation of drug efflux pumps (such as ABC transporter family), the enhancement of DNA repair mechanisms, and the evasion of apoptosis (Gu et al., 2025; Nesic et al., 2025). Evidence shows that autophagy gets rid of faulty organelles and proteins, allows cancer cells to survive the effects of chemotherapy by clearing out accumulated cytotoxic substances (Ivanova et al., 2025; Yang et al., 2024a). For example, Tripartite-motif protein 44 (TRIM44), a deubiquitinating enzyme, promotes doxorubicin resistance by enhancing autophagic activity (Wang Y. et al., 2025). Moreover, ubiquitination modulates the stability and activity of key autophagy regulators (Table 1), thereby indirectly shaping autophagic flux and cellular metabolism. Specific E3 ligases that modify these autophagy components can either enhance or suppress the protective autophagic response, affecting tumor chemosensitivity.

**TABLE 1:** Ubiquitination-autophagy axis which regulate cancer chemotherapy resistance.

Regulator	Level	Cancer type	Target/pathway	Autophagy	Therapeutic resistance	Biological Processes	Ref.
TRIM65 (E3 ligase)	↑	NSCLC	TNRC6A→TRIM65/ATG7	Activates	Cisplatin	Ubiquitination Autophagy apoptosis	Pan et al. (2019)
miR-135b-5p (miRNA)	↑	Colorectal cancer	ULK1→miR-135b-5p/MUL1	Activates	Oxaliplatin	Ubiquitination autophagy	Wang H. et al. (2021)
TRIM28 (E3 ligase)	↑	HCC	HMGB1→HN1/MAGE-A3/6/TRIM28	Activates	oxaliplatin	Polyubiquitination lysosomal degradation autophagy	Wang R. et al. (2022)
CRL4^CUL4A/DDB1^ (E3 ligase)	↓	Ovarian cancer	CRL4→AMPKα^Thr172^/ MFFSer172/Ser146/DRP1	Activates	Cisplatin	Ubiquitination Proliferation mitophagy	Meng et al. (2022)
DTX2 (E3 ligase)	↑	NSCLC	NCOA4→DTX2/NCOA4	Activates	Cisplatin	Ubiquitination proteasomal degradation ferritinophagy ferroptosis	Liu Z. et al. (2024)
MUL1 (E3 ligase)	↓	HNC	TBK1→MUL1/SG	Activates	Cisplatin	Ubiquitination autophagy	Kim et al. (2025)
Linc00673 (lncRNA)	↑	NSCLC	Smad3→Linc00673/STUB1	Activates	Cisplatin	Ubiquitination autophagy	Ni et al. (2024)
USP49(DUB)	↑	RB	USP49→IGF2BP3/SIRT1	Activates	Carboplatin	Ubiquitination autophagy	Li et al. (2024)
GBCDRlnc1 (lncRNA)	↑	Gallbladder cancer	PGK1→GBCDRlnc1/ATG5–ATG12 complex	Activates	Dox	Ubiquitination proteasomal degradation autophagy	Cai et al. (2019)
TBX15	↑	Breast cancer	PKM2→TBX15/miR-152	Suppresses	Dox	Ubiquitination Autophagy glycolytic flux	Jiang et al. (2021)
MAGEA6 (E3 ligase)	↑	TNBC	AMPKα1→MAGEA6/AMPK	Suppresses	Dox	Ubiquitination proteasomal degradation autophagy	Zhu et al. (2024)
CXCL1(Chemokine)	↑	Breast cancer	IGF1R→CXCL1/VHL/STAT3/HMGB1	Activates	Dox	Ubiquitination proteasomal degradation autophagy	Yang et al. (2024b)
Pritimerin	↑	TNBC	HSPA8→VAV1/GEF/ERK	Suppresses	Dox	Ubiquitination proteasomal degradation autophagy	Liu Q. W. et al. (2025)
CaO_2_-MNPs	↑	TNBC	HIF-1α→CaO2-MNPS/PHD/VHL	Suppresses	Dox	Ubiquitination Autophagy apoptosis	Cheng et al. (2021)
ATG5	↑	NIH 3T3 cells	LC3→ATG5–tATG5	Suppresses	Paclitaxel	Ubiquitination autophagy	Eom et al. (2019)
GRP78 (E3 ligase)	↑	Breast cancer	β-catenin→Akt/GSK3β	Activates	Paclitaxel	Phosphorylation Ubiquitination proteasomal degradation	Liao et al. (2021)
NEDD8 (E3 ligase)	↑	TNBC	TFEB→NEDD8/UBC12/TRIM25	Activates	Paclitaxel	Polyubiquitination nuclear translocation autophagy mitophagy	Zheng et al. (2024)
HAPSTR1	↑	Ovarian cancer	LRPPRC→PSMD14/HAPSTR1	Activates	Paclitaxel	ubiquitination proliferative autophagy	Li and Wang (2024)
HERC3 (E3 ligase)	↑	Glioblastoma	SMAD7→HERC3/TGF-β/SMAD2/3/EMT	Suppresses	Temozolomide	Ubiquitination proteasomal degradation autophagy	Li et al. (2019)
BCYRN1(lncRNA)	↑	NK/T-cell lymphoma	p53→BCYRN1//mTOR/ULK1	Activates	Asparaginase	Ubiquitination autophagy	Wang L. et al. (2021)

TRIM65,Tripartite-motif protein 65; NSCLC, non-small cell lung cancer; TNRC6A,Trinucleotide Repeat Containing 6A; ATG7,Autophagy related 7; ULK1,Unc-51, like autophagy activating kinase 1; MUL1,Mitochondrial E3 ubiquitin ligase 1; TRIM28,Tripartite-motif protein 28; HCC, hepatocellular carcinoma; HMGB1,high mobility group protein B1; HN1,Hematological and neurological expressed 1; MAGE, A3/6,Melanoma-associated antigen3/6; TRIM28,Tripartite-motif protein 28; CRL4,Cullin-RING, Ligase 4; AMPKα,AMP-activated protein kinase; MFF, mitochondrial fission factor; DRP1,dynamin-related protein 1; DTX2,Deltex E3 ligase 2; NCOA4, nuclear receptor coactivator 4; HNC, head and neck cancer; TBK1,TANK, binding kinase 1; SG, stress granule; Smad3,SMAD, family member 3; STUB1,E3 ligase; USP49,Ubiquitin Specific Peptidase 49; IGF2BP3,insulin-like growth factor 2 mRNA-binding protein 3; SIRT1,stabilization of sirtuin 1; GBCDRlnc1,gallbladder cancer drug resistance-associated lncRNA1; PGK1,phosphoglycerate kinase 1; Dox, Doxorubicin; TBX15,T-box transcription factor 15; PKM2,Pyruvate kinase M2; MAGEA6,Melanoma-associated antigen A6; TNBC, Triple-negative breast cancer; CXCL1,C-X-C motif chemokine ligand 1; IGF1R,Insulin-like growth factor 1 receptor; HSPA8,Heat Shock Protein Family A Member 8; VAV1/ERK, Vac Guanine Nucleotide Exchange Factor 1/Extracelluar regulated protein kinases; CaO_2_-MNPs, Calcium Peroxide-Loaded Magnetic Nanoparticles; HIF-1α,hypoxia-inducible factor 1α; PHD, prolyl hydroxylase; VHL, Von Hippel–Lindau; LC3,Microtubule-Associated Protein 1 Light Chain 3; GRP78,Glucose regulated protein78; NEDD8,neural precursor cell expressed developmentally downregulated protein 8; TFEB, transcription factor EB; HAPSTR1,HUWE1 associated protein modifying stress responses; LRPPRC, leucine rich pentatricopeptide repeat containing protein; PSMD14,Proteasome 26S Subunit, Non-ATPase, 14; HERC3,HECT, and RLD, Domain Containing E3 Ubiquitin Protein Ligase 3; BCYRN1,Brain cytoplasmic RNA, 1; mTOR, Mammalian Target Of Rapamycin.

### 2.2 Platinum-based drug resistance: Role of E3 ligases and ubiquitin chain specificity

#### 2.2.1 Introduction of platinum-based drug

Platinum-based drugs, including cisplatin, carboplatin, and oxaliplatin, are widely used as first-line chemotherapeutic agents for solid tumors such as lung, ovarian, colorectal, and testicular cancers (Yang et al., 2025; Zhang et al., 2022). Their cytotoxic effect is primarily mediated through the formation of DNA adducts that trigger DNA damage responses, ultimately inducing apoptosis in rapidly proliferating tumor cells (Ciarimboli, 2021; Sahoo et al., 2024). Although platinum-based drugs are widely used and show strong initial effects, both intrinsic and acquired resistance greatly reduce their long-term success. Growing evidence shows that abnormal ubiquitination is a key factor controlling autophagy during platinum-based chemotherapy. In particular, the ubiquitination of autophagy-related proteins and cargo receptors changes autophagic flux, which affects tumor cell survival under stress caused by cisplatin or oxaliplatin (Cen et al., 2023; Yin et al., 2025).

#### 2.2.2 Ubiquitination–autophagy axis in platinum-based drug resistance

In cisplatin-resistant non-small cell lung cancer (NSCLC), TRIM65 is markedly upregulated while miR-138-5p is significantly downregulated, exhibiting a clear inverse correlation. Mechanistically, TRIM65 promotes K48-linked ubiquitination and subsequent degradation of Trinucleotide Repeat Containing 6A (TNRC6A), which results in a reduction in levels of miR-138-5p and relief of its suppressive effect on the 3’UTR of the autophagy-related gene ATG7. The resulting increase in ATG7 transcription and protein expression enhances LC3-I to LC3-II(Microtubule-Associated Protein 1 Light Chain 3) conversion and autophagosome formation, enabling tumor cells to eliminate cisplatin-induced damage via autophagy, attenuate Caspase-3 activation, and reduce early apoptosis (fewer Annexin V^+^ cells). Conversely, TRIM65 knock-out restores miR–138–5p–mediated repression of ATG7, significantly diminishes autophagic activity, increases cisplatin-induced apoptosis, and suppresses growth of resistant tumors *in vivo* (Pan et al., 2019).

In case of colorectal cancer, the upregulation of miR-135b-5p promotes resistance to the chemotherapeutic agent oxaliplatin by targeting the Mitochondrial E3 ubiquitin ligase 1/Unc-51 like autophagy activating kinase 1 (MUL1/ULK1) axis. Specifically, miR-135b-5p suppresses MUL1, diminishing its role in ubiquitination and degradation of ULK1. The resulting stabilization of ULK1 enhances protective autophagy, thereby enabling tumor cells to withstand the stress induced by oxaliplatin. These findings identify miR-135b-5p as a promising therapeutic target to sensitize colorectal cancer to oxaliplatin (Wang H. et al., 2021).

In hepatocellular carcinoma (HCC), Hematological and neurological expressed 1(HN1) associates with the scaffold protein Melanoma-associated antigen3/6 (MAGE-A3/6) and recruits the E3 ligase Tripartite-motif protein 28 (TRIM28) to inhibit K48-linked polyubiquitination of high mobility group protein B1(HMGB1). This blockade prevents HMGB1’s lysosomal degradation via the autophagy pathway, resulting in its accumulation. Intracellular HMGB1 exerts dual functions: within the nucleus, it drives transcription of DNA damage–repair genes; in the cytoplasm, it activates autophagy, thereby enhancing oxaliplatin resistance. Notably, overexpressing HMGB1 completely reverses the autophagy suppression caused by HN1 loss. This finding suggests that blocking the HN1–TRIM28 interaction or restoring HMGB1-mediated autophagic degradation could be new ways to overcome chemoresistance in HCC (Wang R. et al., 2022). In ovarian cancer, loss of the CRL4^CUL4A/DDB1^ (DNA damage-binding protein 1) complex promotes mitochondrial fission by activating AMPKα^Thr172^ (AMP-activated protein kinase) and MFF^Ser172/Ser146^(mitochondrial fission factor) phosphorylation, which recruits dynamin-related protein 1 (DRP1). This process triggers Parkin–PINK1–mediated mitophagy to remove damaged mitochondria, thereby slowing cell proliferation. Both pharmacological and genetic inhibition of autophagy partially reverse this growth arrest, indicating that CRL4 functions as E3 enzyme to regulate chemosensitivity through a ubiquitin-dependent mitochondrial quality control network (Meng et al., 2022). Deltex E3 ligase 2 (DTX2), an E3 ligase, binds the nuclear receptor coactivator nuclear receptor coactivator 4(NCOA4) and catalyzes its K48-linked ubiquitination and subsequent proteasomal degradation. This downregulates the NCOA4–mediated process known as ferritinophagy and ferroptosis in cell lines derived from non-small-cell lung carcinoma (NSCLC). Consequently, DTX2 knockout enhances cisplatin-induced ferroptotic cell death and overcomes chemoresistance in these cells (Liu Z. et al., 2024). Loss of the mitochondrial E3 ligase MUL1 increases TANK binding kinase 1 (TBK1) activation in head and neck cancer (HNC) cells and tissues. Under stress conditions, activated TBK1 triggers protective autophagy to remove ubiquitinated proteins and promotes stress granule (SG) formation through autophagic processes. These effects enhance cisplatin resistance in HNC cells (Kim et al., 2025). The long noncoding RNA Linc00673 interacts with SMAD family member 3(Smad3), preventing its ubiquitination and subsequent E3 ligase STUB1-mediated degradation, thereby promoting chemoresistance in NSCLC. Concurrently, Linc00673-V3 upregulates LC3B transcription, enhancing autophagic flux and ultimately driving cisplatin resistance in NSCLC cells (Ni et al., 2024). Additionally, Deubiquitinating enzymes can promote chemoresistance by regulating ubiquitin-dependent protein modification. In an m6A-dependent way, insulin-like growth factor 2 mRNA-binding protein 3(IGF2BP3) increases Ubiquitin Specific Peptidase 49(USP49) expression, which prevents the ubiquitin-mediated degradation of stabilization of sirtuin 1(SIRT1) and maintains its function in metabolic control. The resulting accumulation of SIRT1 then activates autophagy, thereby enhancing Retinoblastoma (RB) resistance to CBP (Li et al., 2024).

### 2.3 Doxorubicin resistance: ubiquitin-modulated autophagic regulators

Gallbladder cancer drug resistance-associated lncRNA1 (GBCDRlnc1) binds phosphoglycerate kinase 1(PGK1) and prevents its K48-linked ubiquitination and degradation by the proteasome, which leads to PGK1 accumulation in doxorubicin-resistant gallbladder cancer cells. The stabilized PGK1 then promotes conjugation of the ATG5–ATG12 complex, enhancing autophagosome elongation and thereby driving adaptive resistance to doxorubicin (Cai et al., 2019).

Furthermore, in breast cancer, the TBX15/miR-152/KIF2C axis has been demonstrated to play a key role in the modulation of doxorubicin resistance by regulating the ubiquitination and stability of Pyruvate kinase M2(PKM2). Specifically, T-box transcription factor 15 (TBX15) overexpression induces miR-152, which promotes K48-linked ubiquitination of PKM2, leading to its destabilization; This, in turn, suppresses both autophagy and glycolysis, which re-sensitizes cells to doxorubicin (DOX). In contrast, Kinesin family member 2C(KIF2C) blocks PKM2 ubiquitination, maintains PKM2 levels, restores autophagy and glycolytic activity, thereby increases DOX resistance (Jiang et al., 2021).

The MAGEA6–AMPKα1 axis plays a key role in triple-negative breast cancer (TNBC). Melanoma-associated antigen A6 (MAGEA6) is part of an E3 ligase complex that promotes AMPKα1 ubiquitination and speeds up its degradation by the proteasome. This ubiquitin-dependent reduction of AMPK signaling blocks autophagy and increases chemoresistance. Notably, the autophagy inhibitor chloroquine completely reverses the chemotherapy resistance caused by MAGEA6 loss. This tumor-specific E3 ligase pathway shows that AMPKα1 degradation suppresses autophagy and promotes resistance. The findings suggest a dual-target approach to overcome TNBC chemoresistance by inhibiting MAGEA6 to restore AMPK-dependent autophagy and activate SLC7A11-mediated ferroptosis (Zhu et al., 2024).

C-X-C motif chemokine ligand 1(CXCL1) disrupts the association between the E3 ligase adaptor VHL and the insulin-like growth factor 1 receptor (IGF1R), markedly decreasing IGF1R ubiquitination and blocking its proteasomal degradation. The consequent accumulation of IGF1R aberrantly activates STAT3 signaling, leading to upregulated transcription of high mobility group box 1 (HMGB1). As a pivotal autophagy regulator, HMGB1 enhances autophagic flux by promoting LC3-II/I conversion and reducing p62 levels, thus fostering an ABCG2-dependent drug-efflux phenotype and conferring chemoresistance. Importantly, both *in vitro* and *in vivo* assays confirmed that CXCL1’s stabilization of IGF1R is dose-dependent. This work uncovers a critical cross-talk between chemokine signaling and the ubiquitin system within the tumor microenvironment and provides a rationale for targeting the TAM–CXCL1/IGF1R/STAT3/HMGB1 axis to overcome autophagy-driven resistance (Yang et al., 2024b).

Pristimerin promotes the ubiquitination and degradation of HSPA8, a key protein in chaperone-mediated autophagy (CMA). This process disrupts protein quality control and alters Vac Guanine Nucleotide Exchange Factor 1/Extracelluar regulated protein kinases (VAV1/ERK) signaling, which drives doxorubicin resistance in triple-negative breast cancer (TNBC). Blocking HSPA8 pharmacologically increases the effect of pristimerin by restoring autophagy and making tumor cells more sensitive to doxorubicin. These findings suggest that targeting the ubiquitin–autophagy axis could help overcome chemoresistance (Liu Q. W. et al., 2025).

In TNBC, hypoxia induces doxorubicin resistance by stabilizing hypoxia-inducible factor 1α(HIF-1α) through Prolyl Hydroxylase (PHD) inhibition, which suppresses apoptosis and activates protective autophagy. Oxygen-generating CaO_2_-MNPs restore PHD activity and promote VHL E3 ligase–mediated ubiquitination and degradation of HIF-1α. This attenuates autophagic flux, reinstates apoptosis, and enhances doxorubicin efficacy. Thus, ubiquitination-driven regulation of HIF-1α stability links autophagy to chemoresistance under hypoxic stress (Cheng et al., 2021).

It is evident that these mechanisms collectively emphasize the critical function of ubiquitin–autophagy crosstalk in the process of adaptive chemoresistance and suggest that dual targeting of metabolic and proteolytic pathways may provide new therapeutic opportunities.

### 2.4 Paclitaxel resistance: E3 ligase–dependent autophagy axis in action

ATG5, as a substrate in the ubiquitin-like conjugation system, mediates K48-linked ubiquitination of the autophagy protein LC3. Its deletion impairs autophagosome formation and renders NIH 3T3 cells resistant to paclitaxel, characterized by attenuated G2/M cell-cycle arrest, reduced early apoptosis, and loss of the apoptosis-related truncated ATG5 fragment (tATG5). This resistance is independent of ABC transporter activity. Remarkably, re-expression of the ATG5 N-terminal cleavage product (tATG5) restores paclitaxel sensitivity in knockout cells. These findings indicate that paclitaxel resistance upon ATG5 loss arises from the inability to generate tATG5, revealing a noncanonical role for ATG5 in chemoresistance and suggesting that targeting the ATG5–tATG5 axis may be a strategy that could be employed to overcome resistance to paclitaxel that is driven by the Ras protein (Eom et al., 2019).

Overexpression of Glucose regulated protein78 (GRP78), inhibits the Akt/GSK3β signaling axis, leading to decreased levels of β-catenin phosphorylation and blocks the ubiquitin-dependent proteasomal breakdown of β-catenin. The resulting stabilization of β-catenin promotes its nuclear translocation and interaction with TCF/LEF transcription factors, upregulating the drug efflux pump ABCG2. This dual enhancement of autophagy and drug export drives chemoresistance to paclitaxel in breast cancer cells. In contrast, Ai Du Qing (ADQ) treatment restores β-catenin ubiquitination, facilitating its Akt/GSK3β-mediated proteasomal turnover, downregulating ABCG2, reducing drug efflux, and suppressing autophagy—thereby counteracting GRP78-mediated resistance. These results highlight the therapeutic potential of combining paclitaxel with ADQ and provide a basis for further studying the efficacy of ADQ in reversing drug resistance in other tumor models (Liao et al., 2021).

In paclitaxel-resistant triple-negative breast cancer (TNBC), neural precursor cell expressed developmentally downregulated protein 8(NEDD8) and its conjugating enzyme UBC12 are abnormally activated. UBC12 adds NEDD8 to Lysine at site 117 (Lys117) in the RING domain of the E3 ligase TRIM25, reducing steric hindrance and improving substrate binding. Activated TRIM25 promotes K63-linked polyubiquitination of the transcription factor transcription factor EB (TFEB), which drives its movement into the nucleus and increases the expression of autophagy-related genes such as LC3B and ATG5, as well as mitophagy markers. The resulting rise in autophagy and mitophagy activity lowers tumor cell sensitivity to paclitaxel (PTX). Blocking the NEDD8-activating enzyme with MLN4924, or deleting UBC12 or TRIM25, inhibits TFEB-dependent transcription of autophagy genes and partially restores PTX sensitivity. These findings suggest that co-targeting the UBC12–TRIM25–TFEB pathway with PTX could produce a synergistic effect in suppressing tumor growth and metastasis (Zheng et al., 2024).

HUWE1 Associated Protein Modifying Stress Responses (HAPSTR1), as a direct target of miRNAs associated with paclitaxel resistance. Mechanistically, HAPSTR1 binds to Leucine rich pentatricopeptide repeat containing protein (LRPPRC) and prevents its ubiquitination by recruiting the deubiquitinase PSMD14, thereby inhibiting degradation of LRPPRC. Importantly, knockdown of LRPPRC abolishes the pro-proliferative, invasive, and migratory effects conferred by HAPSTR1’s overexpression in ovarian cancer cells, highlighting the HAPSTR1–PSMD14–LRPPRC axis as a potential promising target to overcome paclitaxel resistance (Li and Wang, 2024).

### 2.5 Other chemotherapeutic agents: non-canonical ubiquitin-autophagy pathways

In glioblastoma (GBM), the autophagy–lysosome pathway increases the expression of the E3 ligase HERC3. The HECT domain of HERC3 catalyzes K63-linked ubiquitination of SMAD7—a negative regulator of TGF-β signaling—through its RCC4–7 interaction module, leading to SMAD7 degradation by the proteasome. The loss of SMAD7 removes its inhibitory effect on the TGF-β/SMAD2/3 pathway, which promotes epithelial–mesenchymal transition (EMT) and temozolomide (TMZ) resistance. Blocking HERC3, either pharmacologically or genetically, reduces GBM cell invasion and EMT, identifying HERC3 as a potential target to overcome TMZ resistance (Li et al., 2019).

Overexpression of Brain cytoplasmic RNA 1 (BCYRN1) enhances polyubiquitination and subsequent degradation of p53, thereby inhibiting the p53/mTOR axis. Releasing mTOR’s inhibition of the autophagy initiator ULK1 enhances protective autophagy and reduces asparaginase (ASP)–induced growth inhibition in extranodal NK/T-cell lymphoma cells. This BCYRN1–ubiquitination–p53/mTOR–ULK1 pathway shows how ubiquitin modifications control autophagy through p53/mTOR signaling and highlights a new target to overcome ASP resistance (Wang L. et al., 2021).

### 2.6 Context-dependent regulation of autophagy by ubiquitination across chemotherapy

Across chemotherapy settings, the impact of autophagy on drug response is clearly context-dependent, and ubiquitination appears to act as a key “tuner” of this effect (Pan et al., 2019; Wang H. et al., 2021; Wang R. et al., 2022; Meng et al., 2022; Liu Z. et al., 2024; Kim et al., 2025; Ni et al., 2024; Li et al., 2024; Eom et al., 2019; Liao et al., 2021; Zheng et al., 2024; Li and Wang, 2024; Li et al., 2019; Wang L. et al., 2021). Depending on tumor type and stress conditions, ubiquitin-controlled substrate selection and chain topology can shift autophagy toward different outputs (global flux versus cargo-selective programs), so the same autophagy machinery may confer resistance in one setting but not in another (Wang H. et al., 2021; Meng et al., 2022; Liu Z. et al., 2024; Kim et al., 2025; Zheng et al., 2024; Li et al., 2019; Wang L. et al., 2021). Overall, these observations argue that chemotherapy-associated “autophagy dependency” is not uniform, and that the relevant ubiquitin–autophagy checkpoint likely differs across tumor contexts, supporting mechanism-matched rather than blanket autophagy-targeting strategies.

## 3 Ubiquitin–autophagy axis in targeted therapy resistance

### 3.1 Introduction of targeted therapy

**FIGURE 2:**
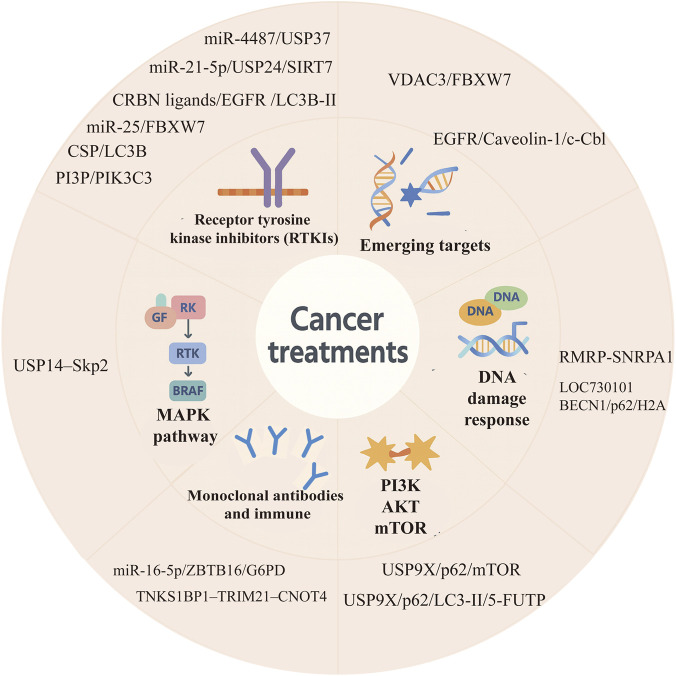
Overview of the main regulatory nodes in targeted cancer therapies involving ubiquitination and autophagy. This schematic shows the key molecular regulators and signaling axes in six major targeted therapy types: receptor tyrosine kinase inhibitors (RTKIs), monoclonal antibodies and immune checkpoint inhibitors, DNA damage response inhibitors, PI3K/AKT/mTOR inhibitors, MAPK pathway inhibitors, and other molecular targets. MiR,miRNA; USP37,Ubiquitin Specific Peptidase 37; USP24,Ubiquitin Specific Peptidase 24; SIRT7,Sirtuin 7; CRBN, cereblon; EGFR, epidermal growth factor receptor; FBXW7,F-box and WD repeat domain-containing 7; CSP, Circumsporozoite protein; PI3P,Phosphatidylinositol 3-phosphate; LC3B,Microtubule-associated protein 1 light chain 3β; PIK3C3,Phosphatidylinositol 3-kinase catalytic subunit type 3; USP14,Ubiquitin Specific Peptidase 14; Skp2,S-phase kinase-associated protein 2; VDAC3,Voltage-Dependent Anion Channel 3; c-Cbl, Casitas B-lineage lymphoma; RMRP, RNase MRP; SNRPA1,Small nuclear ribonucleoprotein polypeptide A; LOC730101,lncRNA Gene; BECN1,Beclin 1; H2A,Histone 2A; ZBTB16,Zinc Finger and BTB Domain Containing 16; G6PD,glucose-6-phosphate dehydrogenase; TNKS1BP1,tankyrasel binding protein 1; TRIM21,Tripartite motif containing 21; CNOT4,CCR4-NOT transcription complex subunit 4; USP9X,Ubiquitin specific peptidase 9 X-linked; mTOR, Mammalian Target Of Rapamycin; 5-FUTP,5-fluorouridine triphosphate.

Targeted therapies, including receptor tyrosine kinase inhibitors (RTKIs), PI3K/AKT/mTOR pathway inhibitors, and DNA damage response modulators such as poly ADP-ribose polymerase (PARP) inhibitors, have substantially reshaped the landscape of precision oncology by selectively disrupting oncogenic signaling networks (Mele et al., 2020). Recent evidence underscores that ubiquitination and autophagy function as pivotal regulators of these adaptive processes (Table 2), influencing protein stability, intracellular trafficking, and metabolic stress responses that collectively determine tumor sensitivity or resistance to targeted therapies (Young et al., 2024; Jain et al., 2023) (Figure 2).

**TABLE 2:** Ubiquitination-autophagy axis which regulate cancer Targeted Therapy resistance.

Regulator	Level	Cancer type	Target/pathway	Autophagy	Therapeutic resistance	Biological function	Ref.
USP37(DUB)	↓	NSCLC	EGFR→ miR-4487/USP37	Activates	Gefitinib	autophagic degradation apoptosis, ubiquitination	Kim et al. (2022)
miR-21-5p (miRNA)	↑	HCC	SIRT7→ miR-21-5p/USP24	Activates	Sorafenib	protective autophagy ubiquitination	Hu et al. (2023)
miR-25 (miRNA)	↑	HCC	mTOR→ miR-25/FBXW7	Suppresses	Sorafenib	autophagy proteasome degradation ubiquitination	Feng et al. (2022)
PROTAC	↓	NSCLC	EGFR→PROTAC/CRBN ligands	Activates	TKI	autophagy/lysosomal degradation/ubiquitination	Qu et al. (2021)
CSP	↑	LUAD	LC3B→CSP/EGFR-TKIs	Suppresses	TKI	ubiquitin-proteasome/ubiquitin-autophagy-lysosome degradation	Lu et al. (2022)
PI3P	↑	LUAD	PIK3C3→PI3P/EGFR	Activates	Osimertinib	autophagy EGFR endocytosis, ubiquitination	Wang et al. (2023)
NEDD4L	↑	NSCLC	Rab7/VTA1→EGFR	Activates	Gefitinib	recycling of EGFR autophagy, ubiquitination	Wu P. S. et al. (2023)
HUWE1 (E3 ligase)	↑	RCC	MCL-1→ HUWE1/Beclin-1	Activates	Sunitinib	autophagy apoptosis, ubiquitination	Liu T. et al. (2024)
RNF38(E3 ligase)	↑	AML	LMX1A→ RNF38/MG132	Activates	Gefitinib	autophagy proteasomal degradation ubiquitination	Pan et al. (2024)
FTO	↑	NSCLC	PELI3→ RNA m6A/FTO	Activates	Gefitinib	autophagy m6A modification ubiquitination	He et al. (2024)
NEDD8 (E3 ligase)	↑	Liver cancer	p27→CUL1/SCF/NBR1	Activates	Sorafenib	ubiquitin-lysosome degradation autophagy	Xu et al. (2025)
VHL	↑	RCC	ERRα→ p300/CBP/LAMP2/VAMP8	Activates	Sunitinib	autophagy ubiquitin-lysosome degradation	Feng et al. (2025)
UbcH5b	↑	TNBC	p62→ UbcH5b/HECTD3/RNF126	Activates	Lapatinib	autophagy mitochondrial apoptosis ubiquitination	Huang et al. (2021)
miR-16-5p	↑	breast cancer	G6PD→ ZBTB16/miR-16-5p	Activates	Pyrotinib	autophagy, ubiquitination	Liu X. et al. (2023)
TRIM21	↑	HCC	CNOT4→ JAK2/STAT3	Activates	immune	autophagy immune evasion ubiquitination	Wang et al. (2024)
RMRP (lncRNA)	↑	CRC	p53→RMRP/SNRPA1	Activates	PARP inhibitors	nuclear retention ubiquitination, autophagy	Chen et al. (2021)
LOC730101(lncRNA)	↑	Ovarian cancer	H2A→ LOC730101/BECN1/p62	Suppresses	PARP inhibitors	autophagy autophagosome formation ubiquitination	Zhong et al. (2024)
USP9X (DUB)	↑	RCC	K345→ mTOR signaling	Suppresses	Rapamycin	ubiquitination, autophagy	Roldán-Romero et al. (2023), Chen et al. (2023)
USP14 (DUB)	↑	melanoma	Skp2→USP14/Skp2	Suppresses	Vemurafenib	autophagy apoptosis, deubiquitination	Wu T. et al. (2023)
FBXW7 (E3 ligase)	↓ by rapamycin	Lymphoblastic Leukemia	VDAC3→ VDAC3/FBXW7	Activates	Erastin	ubiquitin-proteasomal degradation autophagy lipid peroxidation ferroptosis	Zhu et al. (2021)
	↑by chloroquine						
C-Cbl (E3-ligase)	↑	breast cancer	EGFR→ EGFR signaling axis	Activates	Proguanil	ubiquitin-autophagy-lysosome degradation	Xiao et al. (2022)

USP37,Ubiquitin Specific Peptidase 37; NSCLC, non-small cell lung cancer; EGFR, epidermal growth factor receptor; HCC, hepatocellular carcinoma; SIRT7,Sirtuin 7; FBXW7,F-box and WD, repeat domain-containing 7; PROTAC, proteolysis targeting chimeras; CRBN, cereblon; TKI, tyrosine kinase inhibitors; CSP, circumsporozoite protein; LUAD, lung adenocarcinoma; LC3B,Microtubule-associated protein 1 light chain 3β; PI3P,Phosphatidylinositol 3-phosphate; PIK3C3,Phosphatidylinositol 3-kinase catalytic subunit type 3; NEDD4L,NEDD4 like E3 ubiquitin protein ligase; Rab7,Ras-Related Protein Rab-7a; VTA1,Vesicle trafficking 1; HUWE1,HECT, UBA, And WWE, Domain Containing E3 Ubiquitin Protein Ligase 1; RCC, renal cell carcinoma; RNF38,Ring Finger Protein 38; AML, acute myeloid leukemia; LMX1A,LIM, homeobox transcription factor 1 alpha; MG132,Z-Leu-Leu-Leu-al; FTO, Fat Mass and Obesity-Associated Protein; CUL1,Cullin 1; SCF, E3 ligase complex; NBR1,an autophagy receptor; VHL, E3 ubiquitin ligase; LOC730101, lncRNA; ERRα, Estrogen-related receptor α; CBP,CREB-binding protein; LAMP2,Lysosomal-Associated Membrane Protein 2; VAMP8,Vesicle associated membrane protein 8; UbcH5b,Ubiquitin-conjugating Enzyme H5b; HECTD3,E3 ubiquitin ligase; TRIM21,Tripartite motif containing 21; G6PD, glucose-6-phosphate dehydrogenase; ZBTB16,Zinc Finger and BTB, Domain Containing 16; CNOT4,CCR4-NOT, transcription complex subunit 4; JAK2,Janus kinase 2; STAT3,Signal transducer and activator of transcription 3; RMRP, RNase MRP; SNRPA1,Small nuclear ribonucleoprotein polypeptide A; PARP, poly ADP-ribose polymerase; BECN1,Beclin 1; USP9X,Ubiquitin Specific Peptidase 9X; Skp2,S-phase kinase-associated protein 2; VDAC3,Voltage-Dependent Anion Channel 3; c-Cbl, Casitas B-lineage lymphoma.

### 3.2 Receptor tyrosine kinase inhibitors (RTKIs): EGFR degradation and adaptive autophagy

The key miR-4487/USP37 regulatory axis, which regulates the epidermal growth factor receptor-tyrosine kinase inhibitors (EGFR-TKI) response in non-small cell lung cancer (NSCLC). MiR-4487 targets the deubiquitinase USP37, enhancing gefitinib-induced ubiquitination and autophagic degradation of EGFR, thereby overcoming drug resistance. Importantly, the sensitivity of EGFR to this autophagy-dependent clearance depends on its mutation status: gefitinib effectively promotes EGFR degradation in DELE746-A750-mutated cells, but is ineffective in wild-type or T790M mutation models. Gefitinib treatment downregulates USP37, increases the level of miR-4487, increases EGFR ubiquitination and autophagic flux, and ultimately leads to increased apoptosis and decreased drug resistance (Kim et al., 2022). The miR-21-5p/USP24/SIRT7 axis regulates sorafenib resistance in hepatocellular carcinoma (HCC). MiR-21-5p stabilizes Sirtuin 7(SIRT7) protein expression by targeting the deubiquitinating enzyme USP24, thereby activating the protective autophagy pathway and ultimately leading to targeted therapy resistance (Hu et al., 2023). The miR-25/FBXW7 axis. Specifically, sorafenib therapy upregulates miR-25 in HCC cells, directly inhibiting the E3 ligase F-box and WD repeat domain-containing 7(FBXW7), suppressing the ubiquitination modification of mTOR mediated by FBXW7 and thereby reducing FBXW7-mediated ubiquitination and degradation of mTOR, stabilizing mTOR and suppressing autophagy initiation, which contributes to sorafenib resistance (Feng et al., 2022).

In EGFR-mutated non-small cell lung cancer, PROTAC (such as SIAIS125 and SIAIS126) can mediate the dual degradation mechanism of the ubiquitin-proteasome pathway and the ubiquitin-autophagy-lysosome system to overcome tyrosine kinase inhibitor (TKI) resistance, by linking CRBN ligands (polomalidomide) and EGFR inhibitors (canetinib), making EGFR mutations ubiquitinated and degradable while preventing cancer cells from growing. In addition, the autophagy-related biomarker LC3B-II’s translation was activated. Experiments demonstrated that the autophagy contributors rapamycin could greatly enhance PROTAC’s EGFR degradation efficiency and the apoptosis rate, confirming the synergistic effect of the autophagy/lysosomal pathway (Qu et al., 2021).

Circumsporozoite protein, or CSP, promotes the ubiquitin-proteasome pathway’s breakdown of LC3B, which suppresses autophagy and enhances the efficacy of EGFR-TKIs against lung adenocarcinoma (LUAD) (Lu et al., 2022).

Cezanne can promote the expression of Phosphatidylinositol 3-phosphate (PI3P) by inhibiting the ubiquitination modification at the K48 position of Phosphatidylinositol 3-kinase catalytic subunit type 3(PIK3C3) and promoting the transcription of PIK3C3, thereby increasing autophagosome formation and promoting targeted transport of EGFR endocytosis. Finally, it enhances the degradation of autolysosomes and makes lung adenocarcinoma more resistant to Osimertinib (Wang et al., 2023). Non-small cell lung cancer cells with the EGFR T790M mutation enrich endosomal transport factors (e.g., Rab7, VTA1) and the E3 ligase NEDD4L, which together disrupt Ras-Related Protein Rab-7a (Rab7)–EGFR interaction. This impairs Rab7‐dependent recycling of EGFR to the plasma membrane, reroutes the receptor toward lysosomal compartments, and drives its autophagy‐mediated degradation, leading to diminished cell‐surface EGFR signaling and a marked increase in gefitinib resistance (Wu P. S. et al., 2023).

Sunitinib has a dual function: When used alone, it can activate incomplete autophagy, thereby causing drug resistance in renal cell carcinoma. However, when combined with Nilotinib, Nilotinib can activate the E3 ligase HUWE1, and when used in combination, it can significantly enhance the polyubiquitination of the K48 link of Myeloid cell leukemia 1 (MCL-1). Meanwhile, it reduces the phosphorylation at the Thr163 site, increasing the breakdown of the anti-apoptotic protein MCL-1 through the proteasome pathway, thereby weakening MCL-1’s anti-apoptotic effect. After degradation, it releases Beclin-1 bound to it, induces complete autophagic flux and improves the anti-cancer impact. Knockout of MCL-1 can significantly boost Renal Cell Carcinoma (RCC) cells' responsiveness to sunitinib, while HUWE1 knockout completely blocks the promoting effect of the combination of the two drugs on the ubiquitination and degradation of MCL-1 (Liu T. et al., 2024).

Ring Finger Protein 38 (RNF38), a RING-finger E3 ligase, catalyzes K48-linked ubiquitination of the transcription factor LIM homeobox transcription factor 1 alpha (LMX1A), targeting it for proteasomal degradation and thereby reducing LMX1A protein levels. Loss of LMX1A relieves its suppressive effects on autophagy, tumor growth, and gilteritinib sensitivity in acute myeloid leukemia (AML) cells, promoting autophagy activation and driving resistance to gilteritinib. Proteasome inhibition with MG132 prevents LMX1A degradation, while RNF38 knockout or treatment with the autophagy inhibitor 3-MA markedly attenuates both autophagic flux and drug resistance. Together, these results establish the RNF38–LMX1A axis as a critical ubiquitin–autophagy regulatory circuit that modulates AML responsiveness to gilteritinib (Pan et al., 2024).

Overexpression of the m6A demethylase FTO markedly reduces RNA m6A levels in gefitinib-sensitive PC9 cells and induces drug resistance, whereas FTO silencing restores m6A modifications in PC9-GR (gefitinib-resistant) cells and reverses the resistant phenotype. Critically, this modulation requires the E3 ligase PELI3: PELI3 knockdown significantly diminishes resistance and reverses the enhanced resistance conferred by FTO overexpression, while PELI3 overexpression counteracts the sensitizing effect of FTO depletion. Mechanistically, FTO and PELI3 cooperate to activate protective autophagy via the m6A–PELI3 axis, driving gefitinib resistance. These findings identify the FTO–m6A–PELI3–autophagy pathway as a dual-targeted strategy to overcome EGFR-TKI resistance in NSCLC (He et al., 2024).

In sorafenib-resistant liver cancer, NEDD8-mediated neddylation of CUL1 activates the SCF E3 ligase complex, which catalyzes K29-linked ubiquitination of the cyclin-dependent kinase inhibitor p27. These K29 ubiquitin chains serve as a signal for the autophagy receptor NBR1, directing p27 to the autophagosome–lysosome pathway for degradation. P27 deletion interferes with the G1/S cell cycle control, thereby contributing to sorafenib resistance. This CUL1–SCF–p27–NBR1 axis highlights a neddylation-driven ubiquitin–autophagy circuit that underlies acquired resistance to targeted therapy (Xu et al., 2025).

VHL-mutant mutations foster a hypoxic tumor microenvironment that activates the histone acetyltransferases p300/CBP to acetylate Estrogen-related receptor α(ERRα). This acetylation marks ERRα for Parkin‐mediated K48‐linked polyubiquitination and proteasomal degradation. Clearance of ERRα relieves its repression of autophagy‐related gene transcription, thereby upregulating Lysosomal-Associated Membrane Protein 2 (LAMP2) and Vesicle associated membrane protein 8(VAMP8) and promoting autophagosome–lysosome fusion. The net effect is enhanced autophagic flux and increased sunitinib resistance in renal cell carcinoma (Feng et al., 2025).

MicroRNAs and ubiquitin-related enzymes jointly regulate autophagy to influence therapy resistance. Their interaction forms complex signaling networks that control treatment response across cancers. Targeting these pathways may enable personalized strategies to overcome adaptive resistance.

### 3.3 Monoclonal antibodies and immune checkpoint inhibitors: autophagy rewiring via ubiquitin signaling

Lapatinib, a dual EGFR/HER2 inhibitor, promotes recruitment of the E2 enzyme UbcH5b to E3 ligases such as HECTD3 and RNF126, catalyzing K63-linked polyubiquitination of p62 at Lys420. This modification strengthens p62’s interaction with LC3-II, driving autophagosome formation and autophagy activation. The UbcH5b inhibitor PC3-15 directly binds to UbcH5b, preventing HECTD3-mediated ubiquitination of p62 at K420, thereby blocking autophagic flux. Inhibition of this autophagy response triggers the mitochondrial apoptosis pathway and markedly enhances lapatinib sensitivity in TNBC cells (Huang et al., 2021).

MiR-16-5p functions as an upstream negative regulator of the E3 ligase ZBTB16, and their expression levels are inversely correlated. Upon combined treatment with the HER2-targeted inhibitor pyrotinib and the flavonoid chrysin, ZBTB16 is selectively upregulated. Then, ZBTB16 enhances the K48-linked polyubiquitination and proteasomal degradation of glucose-6-phosphate dehydrogenase (G6PD), which triggers autophagy-associated cell death. These findings nominate the miR-16-5p/ZBTB16/G6PD axis as a promising target to overcome resistance to HER2-directed therapies and deliver unique molecular views for breast cancer precision therapy (Liu X. et al., 2023).

Tripartite motif containing 21 (TRIM21), an intracellular antibody effector, forms a complex with tankyrasel binding protein 1 (TNKS1BP1) to ubiquitinate and degrade CCR4-NOT transcription complex subunit 4(CNOT4), thereby suppressing Janus kinase 2-Signal transducer and activator of transcription 3 (JAK2-STAT3) signaling and facilitating HCC progression and immune evasion. Disruption of TNKS1BP1 restores CNOT4 levels, reactivates the JAK2/STAT3 axis, and increases anti-PD-L1 therapy’s effectiveness. In addition, when TNKS1BP1 inhibition reduced the lipid route, it markedly increased autophagy. These findings highlight the medicinal value of TNKS1BP1–TRIM21–CNOT4 pathway targeting to overcome immune resistance in hepatocellular carcinoma (Wang et al., 2024).

These findings show that how cells handle proteins, especially through processes like ubiquitination and autophagy, can directly affect how well targeted treatments and immune therapies work. Targeting these changes may offer new ways to make treatments work better and longer.

### 3.4 DNA damage response inhibitors: lncRNA–E3 ligase circuits influencing autophagy

The long non-coding RNA RMRP assists in colorectal cancer (CRC) sensitivity to PARP inhibitors by modulating the nuclear retention and functional activity of Small nuclear ribonucleoprotein polypeptide A (SNRPA1). Specifically, RMRP binds to SNRPA1, sequestering it within the nucleus and preventing its degradation via chaperone-mediated autophagy (CMA). This nuclear accumulation facilitates SNRPA1-p53 association, which subsequently encourages MDM2-mediated ubiquitination and p53 proteasomal breakdown, reducing its tumor-suppressive properties in the process. As a result, CRC cells exhibit enhanced resistance to PARP inhibition. Notably, in p53-deficient cells, deletion of RMRP still leads to a modest reduction in cell proliferation, suggesting that RMRP may also regulate tumor growth through p53-independent mechanisms (Chen et al., 2021).

The long non-coding RNA LOC730101 impairs autophagosome formation by inhibiting the phosphorylation of Beclin 1(BECN1), a key regulator of autophagy initiation. This blockade results in the intracellular accumulation of p62, which subsequently functions as a negative regulator of the E3 ubiquitin kinase RNF168. Histone H2A’s ubiquitination is hampered by RNF168 inhibition, which interferes with the DNA damage repair process. Through this mechanism, LOC730101 enhances Ovarian cancer cells' response to PARP inhibitors and cisplatin (Zhong et al., 2024).

Targeting lncRNA–ubiquitin–autophagy axes offers new opportunities to overcome resistance in colorectal and ovarian cancers.

### 3.5 PI3K/AKT/mTOR inhibitors: DUBs and ubiquitin scaffolding in autophagy blockade

Emerging evidence highlights the critical role of the deubiquitinating enzyme USP9X in dictating the response of tumor cells to mTOR inhibition. Mechanistically, USP9X regulates the ubiquitination status of p62 at lysine 345 (K345), a key node in autophagy signaling. Loss or pharmacological inhibition of USP9X promotes enhanced ubiquitination and accelerated degradation of p62, thereby impairing autophagic flux. This autophagy blockade disrupts ribosomal RNA processing through aberrant incorporation of 5-fluorouridine triphosphate (5-FUTP), ultimately triggering apoptosis. Functionally, genetic depletion of USP9X or its inhibition by WP1130 markedly sensitizes renal cell carcinoma and cervical cancer cells to the mTOR inhibitor rapamycin, producing a synergistic antitumor effect. Collectively, these findings underscore USP9X as a critical ubiquitin-modifying scaffold that determines the therapeutic efficacy of PI3K/AKT/mTOR pathway inhibitors via modulation of autophagy (Roldán-Romero et al., 2023; Chen et al., 2023).

### 3.6 MAPK pathway inhibitors: Skp2/USP14-mediated autophagy suppression

The USP14–Skp2 axis has been identified as a crucial autophagy regulator that plays a role in the formation of resistance to BRAF inhibitors in melanoma. Mechanistically, USP14 stabilizes Skp2 by deubiquitinating its lysine 119 (K119) residue, thereby preventing its proteasomal degradation. This stabilization enhances Skp2-mediated suppression of autophagy. Importantly, this combinatorial approach has been validated in patient-derived xenograft (PDX) models of acquired BRAF inhibitor resistance, underscoring its translational potential for overcoming therapeutic resistance in melanoma (Wu T. et al., 2023). Dual inhibition of USP14 and Skp2 restores autophagic flux and enhances therapeutic response, offering a promising strategy to overcome vemurafenib resistance.

### 3.7 Other axis in targeted therapy

FBXW7 is an E3 ubiquitin kinase that regulates the proteasomal elimination of VDAC3. Autophagy activator rapamycin (Rapa) downregulates FBXW7 protein level through undefined signaling pathways, while autophagy inhibitors such as chloroquine significantly increase FBXW7 expression. Downregulation of FBXW7 attenuates the ubiquitination and subsequent degradation of VDAC3, leading to its accumulation on the mitochondrial membrane. This buildup enhances the efficacy of erastin, a ferroptosis-inducing agent, by promoting lipid peroxidation and ferroptosis. As a result, resistance of Lymphoblastic Leukemia to erastin becomes promoted. This finding highlights a dual-targeting strategy for overcoming targeted therapy resistance, particularly in acute lymphoblastic leukemia (ALL) and potentially other hematologic malignancies—either by augmenting FBXW7-mediated ubiquitination activity or by directly modulating VDAC3 stability. Moreover, the interaction between autophagy and FBXW7 function, especially in the context of rapamycin-induced autophagy, warrants further investigation as a potential combinatorial therapeutic approach (Zhu et al., 2021).

Among emerging therapeutic strategies targeting ubiquitin-regulated autophagy in drug resistance, Proguanil has recently garnered attention for its novel mechanism of action involving the EGFR signaling axis. Specifically, Proguanil targets the epidermal growth factor receptor (EGFR), promoting its binding to Caveolin-1, which makes it easier for the E3 ligase c-Cbl to be recruited. This cascade enhances EGFR ubiquitination and lysosome-mediated degradation. The downregulation of EGFR and its downstream effectors subsequently triggers autophagy induction in breast cancer cells (Xiao et al., 2022).

These pathways reveal the possibility of focusing on E3 ligases and immune escape regulators to enhance responsiveness across multiple cancer types.

### 3.8 Context-dependent regulation of autophagy by ubiquitination across targeted therapy

Across targeted therapy, autophagy shows a clear context-dependent association with resistance: in receptor tyrosine kinase inhibitor (RTKI) models, autophagy can either support survival by clearing therapy-induced stress signals or contribute to sensitivity by promoting turnover of key signaling proteins, and this opposite outcome is shaped by ubiquitination through chain type and substrate selection that tunes autophagic flux (Kim et al., 2022; Hu et al., 2023; Feng et al., 2022; Qu et al., 2021; Lu et al., 2022; Wang et al., 2023; Wu P. S. et al., 2023; Liu T. et al., 2024; Pan et al., 2024; He et al., 2024; Xu et al., 2025; Feng et al., 2025). Similarly, autophagy is often protective under targeted pressure, but in specific contexts it links to autophagy-associated cell death and improves drug response, again reflecting ubiquitin-guided control over which proteins are modified and how the pathway proceeds (Huang et al., 2021; Liu X. et al., 2023). Notably, responses to PI3K/AKT/mTOR pathway inhibition can hinge on ubiquitination-controlled stability of autophagy hubs such as p62, which fine-tunes flux intensity and thereby influences whether autophagy is adaptive or whether flux disruption becomes pro-apoptotic and sensitizing, supporting a context-matched strategy focused on the responsible DUB/E3 checkpoint rather than blanket autophagy inhibition (Roldán-Romero et al., 2023; Chen et al., 2023).

## 4 Radiotherapy resistance via ubiquitin–dependent autophagy control

### 4.1 Introduction of radiotherapy resistance

**FIGURE 3:**
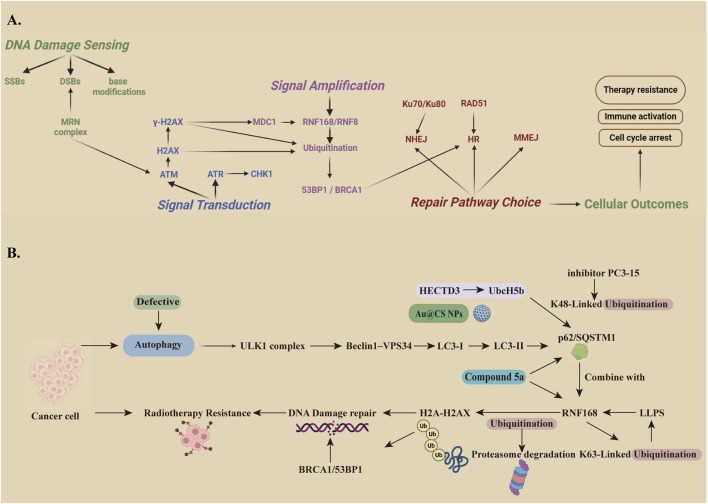
**(A)** Radiotherapy-Mediated DNA Damage Response process: The DNA damage repair (DDR) process after radiotherapy has four stages: damage sensing, signal transduction, signal amplification, and cellular outcome. In the first stage, the MRN complex detects double-strand breaks (DSBs) and brings in the ATM kinase. ATM then phosphorylates histone H2A/H2AX. This step allows MDC1 to recruit RNF168, an E3 ubiquitin ligase that adds ubiquitin to H2A/H2AX. The ubiquitin tag helps 53BP1 and BRCA1 gather at the damaged site and repair the DNA efficiently. SSBs, Single-stranded DNA binding protein; DSBs, double strand breaks; MRN,MRE11-RAD50-NBS1 complex; ATM, ataxia-telangiectasia mutated; H2AX,H2A histone family member X; ATR,ATM and Rad3 related; CHK1,Checkpoint kinase 1; MDC1,Mediator of DNA damage check point protein; RNF168,E3 ubiquitin kinase; RNF8,Ring finger protein 8; BRCA1,Breast cancer susceptibility gene; HR, homologous recombination; RAD51,; NHEJ,Non-homologous end-joining; MMEJ, Microhomology-mediated end joining; **(B)** The Regulatory Mechanism And Influencing Factors Of Ubiquitination-Autophagy Related To Radiotherapy: RNF168 acts as a key E3 ligase that controls chromatin ubiquitination and builds the repair complex. It helps keep DDR working properly and affects how cells respond to radiotherapy and chemotherapy. When autophagy is blocked, or when Compound 5a or the nanodrug Au@CS NPs are used, p62 builds up and inhibits RNF168, which makes cells more sensitive to radiation. In contrast, when the HECTD3–UbcH5b–p62 axis is active, or when K63-linked ubiquitination and RNF168-driven LLPS are increased, tumor cells become more resistant to radiation. The PC3-15 inhibitor can reduce this resistance by blocking the HECTD3–UbcH5b pathway. ULK1,Unc-51 like autophagy activating kinase 1; LC3,Microtubule-associated protein 1 light chain 3; HECTD3,E3 ubiquitin kinase; UbcH5b,Ubiquitin-conjugating Enzyme H5b; SQSTM1,Sequestosome 1; RNF168,E3 ubiquitin kinase; LLPS, liquid–liquid phase separation; H2A,histone 2A; BRCA1,Breast cancer susceptibility gene 1.

Radiotherapy remains a cornerstone in the multidisciplinary management of solid tumors, exerting its antitumor effects primarily through the generation of DNA double-strand breaks and oxidative stress that culminate in cell death (Biau et al., 2019) (Figure 3A). Nevertheless, tumor cells frequently develop resistance by augmenting DNA repair efficiency, remodeling cell cycle checkpoints, and activating survival signaling pathways such as NF-κB and PI3K/AKT (Liu Q. et al., 2025; Wu Y. et al., 2023). Emerging studies have revealed that ubiquitin-dependent signaling and autophagy intersect with these mechanisms critically shaping radiosensitivity and treatment outcome (Yalçin et al., 2024; Yang et al., 2024c) (Table 3).

**TABLE 3:** Ubiquitination-autophagy axis which regulate cancer radiotherapy resistance.

Regulator	Cancer type	Target/pathway	Autophagy	Therapeutic resistance	Biological function	Ref.
Bortezomib	Oral cancer	TRAF6→Akt/mTOR/NF-κB	Activates	Radiotherapy	autophagic degradation, apoptosis, ubiquitination, cell proliferation	Wu et al. (2018)
SIRT3	PINK1/Parkin	H2A→SIRT3/RING1b	Activates	Radiotherapy	ubiquitination, Mitophagy, DNA damage repair	Wei et al. (2023)
RNF168(E3 ligase)	Glioblastoma	Rad51→SQSTM1/p62/RNF168	Activates	Radiotherapy	protective autophagy, ubiquitination	Xu et al. (2022)
RNF168(E3 ligase)	colon cancer	H2A→SQSTM1/p62/RNF168	Activates	Radiotherapy	autophagy, DNA damage repair, ubiquitination	Wang F. C.et al. (2022)
RNF168(E3 ligase)	TNBC	p62→HECTD3/UbcH5b	Activates	Radiotherapy	ubiquination-proteasomal degradation, autophagy,DNA damage repair	Huang et al. (2024)
RNF168(E3 ligase)	multiple cancers	H2A.X→RNF168/53BP1/BRCA1	Activates	Radiotherapy	ubiquination-proteasomal degradation, autophagy,DNA damage repair	Feng et al. (2024)
RNF168(E3 ligase)	autophagy-deficient cells	H2A→SQSTM1/p62/RNF168	Activates	Radiotherapy	ubiquination-proteasomal degradation, autophagy,DNA damage repair	Wang et al. (2017)

TRAF6,tumor necrosis factor receptor-associated factor 6; SIRT3,Sirtuin 3; RNF168,E3 ubiquitin kinase; SQSTM1,Sequestosome 1; HECTD3,E3 ubiquitin kinase; UbcH5b,E2 enzyme; BRCA1,Breast cancer susceptibility gene 1.

### 4.2 Ubiquitin-autophagy axis in radiotherapy resistance

In human oral cancer cells, Bortezomib enhances tumor radiosensitivity by coordinating both ubiquitin- and autophagy-dependent degradation of the pro-survival adaptor TRAF6. It inhibits K63-linked polyubiquitination of TRAF6 and simultaneously promotes its clearance via autophagy. The resulting depletion of TRAF6 disrupts downstream Akt/mTOR and NF-κB signaling, thereby impairing cell proliferation and anti-apoptotic defense mechanisms. Through this dual mechanism, bortezomib effectively reverses radiotherapy resistance and potentiates the efficacy of radiotherapy (Wu et al., 2018).

In colorectal cancer cells, mitochondrial dysfunction induces marked upregulation of Sirtuin 3(SIRT3), which activates PINK1/Parkin-mediated mitophagy. Enhanced mitophagy suppresses RING1b expression, resulting in increased ubiquitination of histone H2A at Lys119. This modification facilitates more efficient repair of radiation-induced DNA damage, thereby conferring enhanced radiotherapy resistance to tumor cells (Wei et al., 2023).

Here, we discuss the key role of RNF168 in radiotherapy resistance (Figure 3B). The autophagy adaptor Sequestosome 1(SQSTM1)/p62 and the E3 ligase RNF168 also play crucial roles in mediating radiotherapy resistance. In glioblastoma, the autophagy adaptor SQSTM1/p62 cooperates with RNF168 to maintain protective autophagy and DNA repair under radiation stress. Core–shell Au@CS nanoparticles disrupt this axis by disrupting lysosomes, thereby blocking autophagic flux with LC3-II and p62 accumulation, while simultaneously enhancing RNF168-mediated ubiquitination and degradation of the DNA repair protein Rad51. This dual inhibition of autophagy and DNA repair sensitizes glioblastoma cells to radiotherapy (Xu et al., 2022).

Compound 5a directly binds to SQSTM1/p62 and RNF168, promoting their interaction and attenuating the ubiquitin ligase activity of RNF168. This inhibition reduces H2A ubiquitination, compromises DNA damage repair, and sensitizes HCT-116 colon cancer cells to radiotherapy (Wang F. C. et al., 2022).

In addition, other E3 ligases can also regulate tumor radiotherapy resistance by mediating the E3 ligase RNF168. The HECTD3–UbcH5b–p62 axis represents a pivotal regulator of radiosensitivity in TNBC. Under physiological conditions, HECTD3 acts as E3 ligase cooperates with the E2 enzyme UbcH5b to catalyze K48-linked ubiquitination of p62, promoting its proteasomal degradation and preventing nuclear accumulation. Loss of HECTD3 abrogates this ubiquitination, causing irradiation-induced p62 nuclear sequestration, which interferes with RNF168-mediated DNA damage repair and enhances tumor cell survival. Pharmacological inhibition of HECTD3/UbcH5b with PC3-15 restores p62 turnover, effectively blocks DNA repair, and significantly increases radiosensitivity (Huang et al., 2024).

RNF168 undergoes liquid–liquid phase separation (LLPS) upon irradiation, driven by K63-linked polyubiquitination chains. This condensation relies on an intrinsically disordered region (aa 460–550) and markedly enhances RNF168-mediated H2A ubiquitination, forming a positive feedback loop that facilitates recruitment of repair factors such as 53BP1 and BRCA1 (Feng et al., 2024).

Autophagy protects RNF168-mediated DNA damage repair by degrading accumulated SQSTM1/p62. In autophagy-deficient cells, p62 directly binds RNF168 via its LIM-binding domain, inhibiting RNF168 catalytic activity and reducing H2A ubiquitination. This impairment blocks the recruitment of key repair factors, including BRCA1, RAP80, and RAD51, to DSB sites, thereby compromising repair efficiency. Consequently, autophagy-deficient cells display persistent γ-H2AX foci, impaired DNA repair, and enhanced radiosensitivity (Wang et al., 2017).

In radiotherapy, autophagy shows clear context dependence: it can protect tumor cells by supporting DNA damage repair and survival signaling, but disrupting autophagic flux can also sensitize tumors by weakening repair capacity and promoting cell death. Ubiquitin-dependent regulation is a key determinant of this bidirectional effect, and these outcomes demonstrate how autophagy and ubiquitin signaling interact in shaping tumor radiosensitivity. Targeting E3 ligases such as HECTD3 or pathways like PINK1/Parkin-mediated mitophagy may offer new strategies to overcome radiotherapy resistance across diverse cancer types.

## 5 Ubiquitin–autophagy signaling regulates immune resistance

### 5.1 Introduction of immune therapy

Immunotherapies, most notably immune checkpoint inhibitors (ICIs) targeting PD-1/PD-L1 and CTLA-4, have achieved unprecedented and durable clinical responses across diverse malignancies, representing a change in cancer treatment (Sharma et al., 2017). However, a significant proportion of patients experience primary refractoriness or acquire resistance during treatment, which is attributed to defective antigen presentation, T cell exhaustion, and tumor-intrinsic immune evasion strategies (Sharma et al., 2023; Borghaei et al., 2021). The evidence shows that ubiquitination and autophagy are essential in these processes (Table 4). They regulate the stability of immune checkpoint proteins, control the autophagic degradation of tumor antigens, and shape the immunogenicity of the tumor microenvironment, thereby influencing the response to immunotherapy (Zhou and Sun, 2021; Hong et al., 2024).

**TABLE 4:** Ubiquitination-autophagy axis which regulate cancer immune therapy resistance.

Regulator	Cancer type	Target/pathway	Autophagy	Therapeutic resistance	Biological function	Ref.
KLHL20–Cul3–ROC1 (E3 ligase)	multiple myeloma	DAPK→KLHL20/IFN-α/γ	Activates	Immune	ubiquitination, apoptosis, autophagy, immunity	Lee et al. (2010)
CPT	Multiple myeloma, breast cancer	TRAF6-ASK1	Activates	Immune	ubiquitination, autophagy, immunity	Yen et al. (2022)
Chlorambucil	ovarian cancer, melanoma	PD-L1→GSK3β/β-TRCP	Activates	Immune	ubiquitination-proteasome degradation, autophagy, immunity	Bai et al. (2023)
Trim35 (E3 ligase)	NSCLC	LSD1→Trim35/ERGIC1/IFNGR1	Suppresses	Immune	ubiquitination, autophagy, immune surveillance	Tang et al. (2023)
USP14 (DUB)	pancreatic cancer	PD-L1→IL-6-USP14	Suppresses	Immune	deubiquitination, autophagy, immune evasion	Zhang et al. (2024)
UBE2T (E2 ligase)	TNBC	CDC42→UBE2T/CD276	Suppresses	Immune	ubiquitination, autophagy, immune evasion	Shi et al. (2025)

DAPK, Death-associated protein kinase; CPT, cryptotanshinone; TRAF6,tumor necrosis factor receptor-associated factor 6; ASK1,apoptosis signal-regulating kinase 1; NSCLC, non-small cell lung cancer; LSD1,lysine-specific histone demethylase 1A; NSCLC, non-small cell lung cancer; ERGIC1,endoplasmic reticulum [ER]-Golgi intermediate compartment 1; IFNGR1,IFN-γ, receptor; PD-L1, Programmed cell death ligand 1; IL-6, interleukin 6; UBE2T,E2 ligase; TNBC, triple-negative breast cancer.

### 5.2 Ubiquitin–autophagy signaling in immune therapy

Interferons (IFNs) can cause autophagic cell death, depending on the cell type. Death-associated protein kinase (DAPK) plays a key role in IFN-induced autophagy. Studies in MCF7 cells show that IFN-γ stops Kelch-like protein 20(KLHL20) from ubiquitinating and degrading DAPK. This block makes DAPK more stable and active. The stabilized DAPK helps form autophagosomes. This is seen as more GFP-LC3 puncta and higher LC3-II levels. These effects disappear when DAPK is lost or when KLHL20 is overexpressed. The stronger autophagy does not come from blocked autophagic flux. It comes from more autophagosome formation. These results show that reducing KLHL20-driven ubiquitination of DAPK supports IFN-induced autophagy (Lee et al., 2010).

Cryptotanshinone (CPT) may act as a new form of immunotherapy by targeting tumor-associated macrophages (TAMs). CPT controls the TRAF6–ASK1 pathway, which is closely linked to ubiquitination. TRAF6 is an E3 ubiquitin ligase that activates ASK1 through K63-linked ubiquitination. This step affects stress and survival signals in the tumor microenvironment. When CPT blocks TRAF6-mediated ubiquitination, it restores the balance of TAM metabolism. It also boosts autophagy, which clears damaged organelles. As a result, macrophages regain their ability to fight tumors (Yen et al., 2022).

In ovarian cancer and melanoma, Chlorambucil boosts anti-tumor immunity by driving PD-L1 degradation through a ubiquitin-dependent process. It activates GSK3β, which helps the E3 ligase β-TRCP add K48-linked ubiquitin chains to PD-L1. This tagging sends PD-L1 to the proteasome for degradation. As PD-L1 levels drop, mTORC1 signaling slows, and autophagy starts. This is shown by higher LC3-II levels and fewer tumor-initiating cells (TICs). These changes support immunogenic cell death. At the same time, PD-L1 loss increases tumor antigen formation, making cancer cells easier for the immune system to find. Natural killer (NK) cells become more active and can destroy tumor cells more effectively. This two-way effect helps reverse immune resistance (Bai et al., 2023).

**FIGURE 4:**
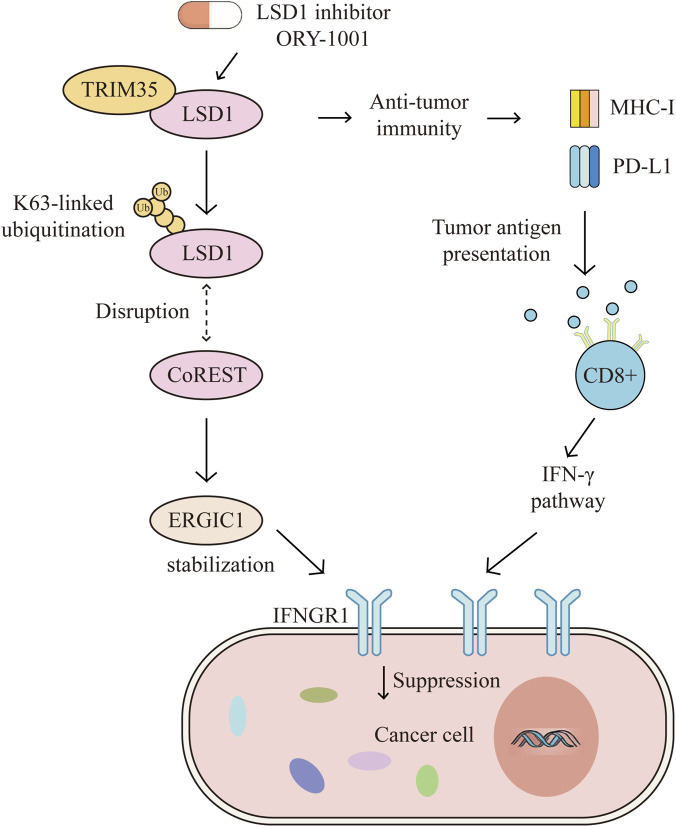
Trim35 mediates K63-linked ubiquitination of LSD1, suppressing its demethylase activity and promoting ERGIC1 transcription. Increased ERGIC1 stabilizes IFNGR1, enhances IFN-γ signaling, and elevates MHC-I expression, thereby improving immune surveillance and sensitizing NSCLC to anti–PD-therapy. In addition, NSCLC cells lacking Trim35 are more responsive to LSD1 inhibitors such as ORY-1001. These findings identify Trim35 as a possible biomarker to guide patient selection for LSD1-targeted immunotherapy. LSD1,lysine-specific histone demethylase 1A; TRIM35,Tripartite-motif protein 35; CoREST, Corepressor for Element-1-Silencing Transcription Factor; ERGIC1,endoplasmic reticulum [ER]-Golgi intermediate compartment 1; IFNGR1,IFN-γ receptor 1; MHC-1,major histocompatibility complex 1; PD-L1,Programmed cell death ligand 1.

In NSCLC, the – – – –IFN-γ receptor (Trim35–LSD1–ERGIC1–IFNGR1) pathway plays a key role in controlling anti-tumor immunity (Figure 4). Trim35 adds K63-linked ubiquitin to LSD1 at lysine 422, which blocks its demethylase activity. When LSD1 is inhibited, ERGIC1 transcription increases. This change lowers autophagy and keeps IFNGR1 stable, which strengthens IFN-γ signaling. As a result, major histocompatibility complex 1 (MHC-I) expression rises, and immune surveillance in NSCLC cells improves. When LSD1 inhibitors are combined with anti–PD-1 therapy, poorly immunogenic lung cancers with low Trim35 levels can be effectively cleared. NSCLC cells without Trim35 also respond better to LSD1 inhibitors such as ORY-1001. These results suggest that Trim35 may serve as a marker for LSD1 activity and help guide personalized treatment strategies that target LSD1 (Tang et al., 2023).

In immunocompetent mouse models, blocking autophagy in cancer-associated fibroblasts (CAFs) lowers interleukin 6(IL-6) levels. This change breaks down the dense stroma in pancreatic cancer and weakens the link between tumor cells and fibroblasts. As a result, STAT3 activity in cancer cells decreases, leading to lower USP14 expression. With less USP14, the deubiquitination of PD-L1 at lysine 280 (K280) is reduced. This allows PD-L1 to build up on the tumor cell surface, making the tumor more sensitive to immune checkpoint therapy and improving anti-tumor immunity (Zhang et al., 2024).

In breast cancer, Ubiquitin-conjugating enzyme E2 T (UBE2T) is highly expressed and plays a key role in immune escape and brain metastasis (BrM). It regulates the UBE2T/CDC42/CD276 pathway through a ubiquitination-dependent process. UBE2T directly binds to CDC42 and promotes its K48-linked polyubiquitination, leading to CDC42 degradation by the proteasome. The loss of CDC42 prevents it from mediating CD276 degradation through the autophagy–lysosome pathway, resulting in increased CD276 levels. High CD276 expression weakens CD8^+^ T-cell function, allowing tumor cells to evade immune surveillance and promoting brain metastasis in triple-negative breast cancer (TNBC). Blocking UBE2T restores immune cell activity and improves the response of TNBC to CD276 immune checkpoint therapy (Shi et al., 2025).

In immune therapy, autophagy is strongly context-dependent: it can support antitumor immunity by promoting immunogenic stress responses and facilitating immune-mediated killing, yet it can also contribute to immune escape by shaping checkpoint protein stability and the tumor microenvironment. Together, these findings underscore the importance of the ubiquitin–autophagy network in shaping tumor immunity and provide a rationale for developing personalized, combination-based immunotherapies to overcome resistance and improve clinical outcomes

## 6 Context-dependent regulation of autophagy by ubiquitination across cancer therapies

Across chemotherapy, targeted therapy, radiotherapy, and immunotherapy, autophagy should be interpreted as a context-dependent stress-response output rather than a uniformly pro-resistance program, with ubiquitination functioning as a multi-layer “rheostat” that calibrates autophagy amplitude, cargo selectivity, and downstream fate decisions across treatment contexts (Ivanova et al., 2025; Yang et al., 2024a; Young et al., 2024; Jain et al., 2023; Yalçin et al., 2024; Yang et al., 2024c; Zhou and Sun, 2021; Hong et al., 2024).

Under chemotherapy, ubiquitin-regulated circuits can elevate cytoprotective autophagic flux to buffer therapy-induced damage and suppress apoptosis—for example, ATG7-centered autophagy activation downstream of TRIM65–miR-138-5p signaling and ULK1 stabilization downstream of the MUL1/ULK1 axis promote survival under platinum stress (Pan et al., 2019; Wang H. et al., 2021). In parallel, selective autophagy programs sustain tolerance by removing damaged organelles or specific substrates, including mitophagy and CMA-linked quality control (Meng et al., 2022; Liu Q. W. et al., 2025). However, chemotherapy-associated autophagy is not unidirectionally protective: it can instead couple to lethal modalities such as ferritinophagy/ferroptosis (where disrupting DTX2–NCOA4 regulation restores ferroptotic killing) and to non-canonical pro-death signaling such as tATG5-dependent cytotoxicity in paclitaxel response, illustrating that outcome depends on stress intensity and cargo/death-pathway coupling (Liu Z. et al., 2024; Eom et al., 2019).

In targeted therapy, pathway blockade and receptor/lysosome trafficking impose proteotoxic and metabolic constraints where autophagy frequently supports persistence and adaptive resistance (e.g., sorafenib- and TKI-resistance programs that are sustained by protective autophagy) (Hu et al., 2023; Liu T. et al., 2024; He et al., 2024; Feng et al., 2025; Wu T. et al., 2023). However, in distinct genetic and signaling contexts, autophagy–lysosome routing can also contribute to treatment efficacy by enabling target clearance and reducing signaling addiction, such as autophagy-dependent EGFR degradation or dual proteasome/autophagy degradation strategies used to overcome EGFR-TKI resistance (Kim et al., 2022; Qu et al., 2021; Wu P. S. et al., 2023; Xiao et al., 2022). Here, ubiquitination determines whether key nodes are stabilized, recycled, or eliminated via proteasome versus autophagy–lysosome routing (Kim et al., 2022; Wu P. S. et al., 2023; Xiao et al., 2022). Chain/site-specific ubiquitin signaling on adaptors and cargo receptors further biases directionality—for example, p62 ubiquitination–dependent flux control shapes drug response, and NBR1-mediated recognition of specific ubiquitin chains can route substrates to autophagic degradation in acquired resistance settings (Xu et al., 2025; Huang et al., 2021; Roldán-Romero et al., 2023; Chen et al., 2023). Moreover, targeted-therapy contexts can include autophagy-associated cell death phenotypes (e.g., G6PD degradation–linked autophagy-associated cell death) and autophagy–ubiquitin crosstalk that modulates ferroptosis response in a context-dependent manner (without implying a single fixed direction across models) (Liu X. et al., 2023; Zhu et al., 2021).

During radiotherapy, resistance is tightly coupled to DNA damage response (DDR) competence, and autophagy can preserve RNF168-driven ubiquitin signaling at DSBs by limiting inhibitory p62 accumulation, thereby sustaining H2A ubiquitination and repair-factor recruitment (Wang et al., 2017). Conversely, radiosensitization can be achieved by therapeutically collapsing autophagic flux and/or attenuating RNF168 activity, including lysosome-disrupting strategies that block autophagy while promoting RNF168-dependent RAD51 loss, or compounds that directly reduce RNF168 ligase activity and H2A ubiquitination (Xu et al., 2022; Wang F. C. et al., 2022). Ubiquitin-dependent control of p62 turnover/localization (e.g., the HECTD3–UbcH5b–p62 axis) further fine-tunes DDR outputs under irradiation (Huang et al., 2024). In addition, irradiation-triggered K63-ubiquitin–driven RNF168 LLPS amplifies H2A ubiquitination and accelerates repair signaling, while mitophagy circuits can also intersect with chromatin ubiquitination and repair efficiency to shape radioresistance (Feng et al., 2024; Wei et al., 2023). A complementary radiosensitizing route is dismantling pro-survival signaling by coordinating ubiquitin- and autophagy-dependent degradation of upstream adaptors such as TRAF6, thereby suppressing Akt/mTOR and NF-κB survival signaling (Wu et al., 2018).

In immunotherapy, ubiquitination and autophagy jointly tune tumor immunogenicity and immune escape in a compartment-specific manner (Sharma et al., 2017; Sharma et al., 2023; Borghaei et al., 2021; Zhou and Sun, 2021; Hong et al., 2024). IFN-linked programs can induce autophagosome formation via stabilization of DAPK when KLHL20-driven ubiquitination is restrained, supporting IFN-induced autophagy in a cell-type–dependent fashion (Lee et al., 2010). Autophagy can also support antitumor immunity through myeloid reprogramming, as exemplified by TRAF6 K63-ubiquitination blockade that restores TAM metabolism and enhances autophagy-mediated organelle quality control (Yen et al., 2022). Ubiquitin-dependent PD-L1 degradation can couple checkpoint attenuation with mTORC1 suppression and autophagy induction, aligning with enhanced immunogenicity and improved immune-cell cytotoxicity (Bai et al., 2023). Conversely, reduced autophagy can be immunostimulatory in specific tumor-intrinsic circuits, such as Trim35-mediated inhibition of LSD1 activity that lowers autophagy, stabilizes IFNGR1, strengthens IFN-γ signaling, and improves MHC-I–mediated immune surveillance with improved response to checkpoint blockade combinations (Tang et al., 2023). Stromal autophagy modulation can indirectly remodel checkpoint control (CAFs → IL-6/STAT3 → USP14), altering PD-L1 deubiquitination and shifting ICI sensitivity, while ubiquitin-driven blockade of autophagy–lysosome turnover of immune modulators (e.g., UBE2T-driven CDC42 loss preventing CD276 autophagy–lysosome degradation) promotes immune escape (Zhang et al., 2024; Shi et al., 2025).

Collectively, these four therapies converge on a single principle: ubiquitination fine-tunes the functional balance of autophagy by controlling substrate stability, ubiquitin-chain topology, adaptor availability/localization, and selective cargo routing, which explains why indiscriminate autophagy modulation can yield heterogeneous or paradoxical outcomes and motivates mechanism-guided combinations stratified by flux state, dominant selective-autophagy engagement, and E3/DUB (or chain-type) dependence in each therapy context (Pan et al., 2019; Wang H. et al., 2021; Xu et al., 2025; Huang et al., 2021; Wu et al., 2018; Wei et al., 2023; Xu et al., 2022; Wang F. C. et al., 2022; Huang et al., 2024; Feng et al., 2024; Wang et al., 2017; Lee et al., 2010; Yen et al., 2022; Bai et al., 2023; Tang et al., 2023; Zhang et al., 2024; Shi et al., 2025).

## 7 Conclusion and future perspective

Therapeutic resistance remains a major challenge in oncology, compromising the long-term efficacy of immunotherapy, radiotherapy, chemotherapy, and targeted therapies. This review summarizes how specific E3 ligases, deubiquitinases (DUBs), microRNAs, and long noncoding RNAs regulate resistance-related pathways through the ubiquitin–autophagy axis. Across multiple therapeutic modalities—including platinum- and doxorubicin-based chemotherapy, EGFR and PI3K/mTOR inhibitors, and immune checkpoint blockade—resistance frequently converges on the dysregulation of autophagy flux, ubiquitin chain remodeling, and lysosome- or proteasome-mediated degradation. These findings highlight that therapeutic targeting of ubiquitin–autophagy crosstalk represents a promising strategy to overcome treatment resistance. Notably, K63-linked ubiquitination—once regarded as non-degradative—has emerged as a crucial regulator of signaling and autophagy initiation, underscoring the potential of selectively modulating ubiquitin chain topology or E3/DUB–substrate interactions for therapeutic precision.

Several recurring mechanistic themes emerge: E3 ligases such as TRIM65, RNF168, and HERC3 modulate autophagic flux and target key regulators (e.g., TNRC6A, Rad51, SMAD7) for degradation, thereby fine-tuning DNA repair, epithelial–mesenchymal transition (EMT), and survival signaling. Deubiquitinases including USP9X and USP37 function as context-dependent modulators of mTOR and EGFR pathways, adjusting autophagy activity and drug sensitivity. Noncoding RNAs—particularly miR-135b-5p, BCYRN1, and LOC730101—govern the expression of E3 ligases and DUBs, introducing an additional epigenetic layer of control. Immunomodulatory axes—including RNF38–LMX1A and UBE2T–CD276—further link ubiquitin regulation to immune evasion and resistance to checkpoint blockade.

Clinically, efforts to modulate autophagy or ubiquitin-linked protein turnover are increasingly being evaluated in combination settings, and emerging targeted protein degraders further support the feasibility of leveraging ubiquitin-dependent mechanisms in patients. Representative recent and ongoing clinical trials are summarized in Table 5: Recent/ongoing clinical evidence targeting autophagy, ubiquitin signaling, or targeted protein degradation. Given the context-dependent, double-edged role of autophagy and the complexity of ubiquitin networks, future studies should prioritize biomarker-guided patient selection, pharmacodynamic monitoring of autophagy flux, and toxicity-aware dosing/scheduling.

**TABLE 5:** Recent/ongoing clinical evidence targeting autophagy, ubiquitin signaling, or targeted protein degradation.

Cancer	Treatment strategy	Agent or regimen	Target	Stage	Refs
Resectable PDAC (pre-op)	Autophagy inhibition (lysosome)	Hydroxychloroquine + gemcitabine + nab-paclitaxel	Pharmacologic lysosomal autophagy blockade to sensitize therapy	phase II NCT01978184	Zeh et al. (2020)
Platinum-resistant epithelial ovarian cancer	Autophagy + lysosome targeting	HCQ + itraconazole	Dual lysosomal homeostasis disruption	phase I + II NCT03081702	Marastoni et al. (2022)
Advanced solid tumors with MAPK/RAS alterations	Upstream autophagy inhibition	Inlexisertib (DCC-3116) ± trametinib/binimetinib/sotorasib	ULK1/2 inhibition to block autophagy initiation, esp. RAS/MAPK tumors	phase I + II NCT04892017	​
Advanced melanoma	Autophagy modulation + ICI	Nivolumab + HCQ (±ipilimumab)	Test whether autophagy inhibition reshapes response to ICIs	phase I + II NCT04464759	​
Pretreated BRAF V600 melanoma	Autophagy inhibition + targeted therapy	Dabrafenib + trametinib + HCQ	Add autophagy inhibitor to overcome adaptive resistance	phase I + II NCT03754179	Mehnert et al. (2022)
Advanced Solid Tumors	Autophagy inhibition	Chloroquine/Hydroxychloroquine± (Carboplatin and Gemcitabine)	Determine the Safety and Tolerability of Autophagy Inhibition	Phase I NCT02071537	Karim et al. (2022)
ER+/HER2- advanced breast cancer	PROTAC (targeted protein degradation)	ARV-471±IBRANCE	assess the safety, tolerability and anti-tumor activity of ARV-471 alone and combination with palbociclib	phase I + II NCT04072952	​
ER+/HER2- advanced breast cancer	PROTAC (targeted protein degradation)	Vepdegestrant (ARV-471) vs. fulvestrant	ER degradation via UPS	phase III NCT05654623	Campone et al. (2025)
Metastatic castration-resistant prostate cancer	PROTAC (targeted protein degradation)	Bavdegalutamide (ARV-110)	AR degradation in mCRPC	phase I + II NCT03888612	​
Relapsed/refractory malignancies	PROTAC (targeted protein degradation)	DT2216 (BCL-xL degrader)	Target anti-apoptotic protein via degradation	phase I NCT04886622	Mahadevan et al. (2025)
Advanced solid tumors (HRR-altered, etc.)	DUB inhibition (ubiquitin signaling)	KSQ-4279 (USP1 inhibitor)	Target DNA repair dependence; potential for PARPi combinations	phase I NCT05240898	Yap et al. (2024)
Solid tumors/lymphomas (WT p53 enriched)	E3–p53 axis (ubiquitin pathway)	Milademetan (MDM2 inhibitor)	Block MDM2–p53 interaction (ubiquitin-linked p53 control)	phase I NCT01877382	Gounder et al. (2023)
Relapsed/refractory multiple myeloma	CRBN E3 modulation (CELMoD)	Mezigdomide (CC-92480)	Next-gen CRBN modulator	phase I + II NCT03374085	​
chronic myelomonocytic leukemia	Ubiquitin-network targeting (cullin–RING ligase regulation)	Pevonedistat (MLN4924)+azacitidine	Inhibition of NEDD8 activation to alter Cullin-RING E3 network	Phase III NCT03268954	Adès et al. (2022)
acute myeloid leukemia (AML) or myelodysplastic syndromes (MDS)	Ubiquitin-network targeting (population pharmacology)	Pevonedistat ± Azacitidine	Early-phase evaluation of exposure–toxicity–activity relationships for a ubiquitin-pathway modulator	phase I NCT02782468	Handa et al. (2022)

BCL-xL, B-cell lymphoma-extra large; BRAF V600, BRAF V600 mutation; CRBN, cereblon; DUB, deubiquitinase; ER, estrogen receptor; HCQ, hydroxychloroquine; HER2−, human epidermal growth factor receptor 2-negative; HRR, homologous recombination repair; ICI, immune checkpoint inhibitor; mCRPC, metastatic castration-resistant prostate cancer; MDM2, mouse double minute 2; NEDD8, neural precursor cell expressed developmentally downregulated protein 8; PDAC, pancreatic ductal adenocarcinoma; PROTAC, proteolysis-targeting chimera; UPS, ubiquitin specific peptidase; WT, wild-type.

Despite recent progress, several major challenges remain. Most current evidence comes from preclinical models that do not fully reflect clinical complexity. The dual role of autophagy—promoting either cell survival or cell death—makes it difficult to design universally effective modulators. The ubiquitination system is also highly complex, as individual ligases or deubiquitinases can exert context-dependent or even opposing effects under different tumor microenvironmental conditions. Emerging ubiquitin-directed approaches (including PROTACs) face practical constraints such as variable E3 ligase availability across tissues, limited tumor exposure, potential on-target toxicity in normal cells, and acquired resistance driven by changes in target binding or the recruited E3 machinery. Tumor heterogeneity further complicates therapy development, since cancers vary in their dependence on specific ubiquitin–autophagy pathways. Finally, dual inhibitors or dual-target combinations often encounter overlapping toxicities and schedule dependence, highlighting the importance of biomarker-guided patient selection, careful dose/schedule optimization, and rigorous safety evaluation in future clinical studies.

Looking forward, several directions may accelerate translation of the ubiquitin–autophagy field. First, artificial intelligence–enabled prediction of E3–substrate interactions (together with ubiquitinome/proteome data) may help map context-specific ubiquitination circuits, prioritize druggable E3–substrate pairs, and support rational degrader design and biomarker discovery.

Second, future therapeutics should move beyond “global autophagy inhibition” toward agents that can more precisely tune protective versus cytotoxic autophagy in a context-dependent manner—for example, by selectively modulating initiation, cargo selection, or lysosome competence to shift the net outcome toward therapeutic sensitization.

Third, the development of selective PROTACs that target either specific E3 ligases or core autophagy regulators (such as autophagy initiation kinases, ubiquitin-binding cargo receptors, or essential autophagy proteins) may enable more precise intervention than broad pathway inhibitors, while emphasizing the need to manage tissue-specific E3 availability, exposure, and on-target toxicity.

In summary, the ubiquitin–autophagy axis represents a promising but still underexplored area in overcoming therapy resistance. Continued research will help clarify its mechanisms, discover reliable biomarkers, and improve therapeutic strategies. These advances are expected to increase treatment precision, reduce side effects, and provide more lasting clinical benefits for patients with resistant cancers.

## Glossary

TRIM65: Tripartite Motif Containing 65
MiR-138-5p: MicroRNA-138-5p
ATG7: Autophagy Related 7
CRL4: Cullin-RING Ligase 4
USP14: Ubiquitin Specific Peptidase 14
Skp2: S-phase kinase-associated protein 2
UPS: Ubiquitin-Proteasome System
ALP: Autophagy-Lysosome Pathway
DUBs: Deubiquitinating Enzymes
TFEB: Transcription Factor EB
EGFR: Epidermal Growth Factor Receptor
HERC3: HECT and RLD Domain Containing E3 Ubiquitin Protein Ligase 3
SMAD7: SMAD Family Member 7
FTO: Fat Mass and Obesity-Associated Protein
PELI3: Pellino E3 Ubiquitin Protein Ligase 3
PROTACs: Proteolysis Targeting Chimeras
ncRNA: Non-coding RNA
ABC transporter: ATP-Binding Cassette transporter
Beclin 1: Beclin 1 Autophagy Related
LC3: Microtubule-Associated Protein 1 Light Chain 3
SQSTM1: Sequestosome 1
NSCLC: Non-Small Cell Lung Cancer
TNBC: Triple-Negative Breast Cancer
TNRC6A: Trinucleotide Repeat Containing 6A
HMGB1: High Mobility Group Box 1
DTX2: Deltex E3 Ubiquitin Ligase 2
NCOA4: nuclear receptor coactivator 4
HNC: Head and Neck Cancer
SG: Stress Granule
RB: Retinoblastoma protein
CXCL1: C-X-C Motif Chemokine Ligand 1
TAMs: Tumor-Associated Macrophages
IGF1R: Insulin-Like Growth Factor 1 Receptor
HSPA8: Heat Shock Protein Family A Member 8
GEF: Guanine Nucleotide Exchange Factor
PHD: Prolyl Hydroxylase
HIF-1α: Hypoxia-Inducible Factor 1-alpha
**CaO**_**2**_ **-MNPs**: Calcium Peroxide-Loaded Magnetic Nanoparticles
VHL: Von Hippel–Lindau
tATG5: Truncated Autophagy Related 5
AQD: Aiduqing
NEDD8: Neural Precursor Cell Expressed, Developmentally Downregulated 8
PTX: Paclitaxel
GBM: Glioblastoma
EMT: Epithelial–Mesenchymal Transition
TMZ: Temozolomide
ASP: Asparaginase
HCC: Hepatocellular Carcinoma
TKI: Tyrosine Kinase Inhibitor
LUAD: Lung Adenocarcinoma
AML: Acute Myeloid Leukemia
CRC: Colorectal Cancer
CMA: Chaperone-Mediated Autophagy
RCC: Renal Cell Carcinoma
5-FUTP: 5-Fluorouridine Triphosphate
PDX: Patient-Derived Xenograft
Rapa: Rapamycin
ALL: Acute Lymphoblastic Leukemia
CPT: Cryptotanshinone
TICs: Tumor-Initiating Cells
NK: Natural Killer (Cell)
CoREST: Corepressor for Element-1-Silencing Transcription Factor
IFNGR1: Interferon Gamma Receptor 1
CAFs: Cancer-Associated Fibroblasts
IL-6: Interleukin-6.
